# Association Between Burst-Suppression Latency and Burst-Suppression Ratio Under Isoflurane or Adjuvant Drugs With Isoflurane Anesthesia in Mice

**DOI:** 10.3389/fphar.2021.740012

**Published:** 2021-09-06

**Authors:** Di Wang, Qingchen Guo, Di Liu, Xiang-Xi Kong, Zheng Xu, Yu Zhou, Yan Su, Feng Dai, Hai-Lei Ding, Jun-Li Cao

**Affiliations:** ^1^ Jiangsu Province Key Laboratory of Anesthesiology, Jiangsu Province Key Laboratory of Anesthesia and Analgesia Application Technology, Xuzhou Medical University, Xuzhou, China; ^2^ Department of Anesthesiology and Perioperative Medicine, The First Affiliated Hospital of Nanjing Medical University, Nanjing, China; ^3^ NMPA Key Laboratory for Research and Evaluation of Narcotic and Psychotropic Drugs, Xuzhou Medical University, Xuzhou, China; ^4^ Department of Anesthesiology Affiliated Hospital of Xuzhou Medical University, Xuzhou, China

**Keywords:** burst-suppression, burst-suppression latency, burst-suppression ratio, correlation, anesthesia, isoflurane

## Abstract

The same doses of anesthesia may yield varying depths of anesthesia in different patients. Clinical studies have revealed a possible causal relationship between deep anesthesia and negative short- and long-term patient outcomes. However, a reliable index and method of the clinical monitoring of deep anesthesia and detecting latency remain lacking. As burst-suppression is a characteristic phenomenon of deep anesthesia, the present study investigated the relationship between burst-suppression latency (BSL) and the subsequent burst-suppression ratio (BSR) to find an improved detection for the onset of intraoperative deep anesthesia. The mice were divided young, adult and old group treated with 1.0% or 1.5% isoflurane anesthesia alone for 2 h. In addition, the adult mice were pretreated with intraperitoneal injection of ketamine, dexmedetomidine, midazolam or propofol before they were anesthetized by 1.0% isoflurane for 2 h. Continuous frontal, parietal and occipital electroencephalogram (EEG) were acquired during anesthesia. The time from the onset of anesthesia to the first occurrence of burst-suppression was defined as BSL, while BSR was calculated as percentage of burst-suppression time that was spent in suppression periods. Under 1.0% isoflurane anesthesia, we found a negative correlation between BSL and BSR for EEG recordings obtained from the parietal lobes of young mice, from the parietal and occipital lobes of adult mice, and the occipital lobes of old mice. Under 1.5% isoflurane anesthesia, only the BSL calculated from EEG data obtained from the occipital lobe was negatively correlated with BSR in all mice. Furthermore, in adult mice receiving 1.0% isoflurane anesthesia, the co-administration of ketamine and midazolam, but not dexmedetomidine and propofol, significantly decreased BSL and increased BSR. Together, these data suggest that BSL can detect burst-suppression and predict the subsequent BSR under isoflurane anesthesia used alone or in combination with anesthetics or adjuvant drugs. Furthermore, the consistent negative correlation between BSL and BSR calculated from occipital EEG recordings recommends it as the optimal position for monitoring burst-suppression.

## Introduction

Drug-induced general anesthesia is characterized by unconsciousness, amnesia, analgesia, and the relative stability of the autonomic, cardiovascular, respiratory, and thermoregulatory systems (Brown et al., 2010; Nicolaou et al., 2012). A scalp electroencephalogram (EEG) obtained during the onset of general anesthesia is associated with the progressive increase of low-frequency and high-amplitude activity (Kiersey et al., 1951; Akeju et al., 2016). In deep anesthesia, the EEG may present a peculiar pattern of activity known as burst-suppression: high-voltage brain activity (bursts) alternates with periods of isoelectric quiescence (suppressions) (Lewis et al., 2013). The burst-suppression is a fundamental characteristic of a deeply inactivated brain and can also reflect states of hypothermia, certain infant encephalopathies, and coma (Stecker et al., 2001; Ohtahara and Yamatogi, 2003). Clinically, burst-suppression ratio (BSR) is commonly used as an important part of anesthesia depth monitoring, previous reports have shown that the BSR negatively correlates with the bispectral index (BIS: a complex EEG parameter which integrates several disparate descriptors of the EEG into a single variable to titrate depth-of-anesthesia. BIS values range from 0 to 100, and the lower value with the deeper anesthesia) when the BSR exceeds the value of 40 (Bruhn et al., 2000). Thus, the BSR has been demonstrated to be a reliable parameter in the assessment of the depth-of-anesthesia and deep anesthesia.

Clinical studies have identified a possible causal relationship between deep anesthesia and negative short- or long-term patient outcomes. The presentation of burst-suppression during surgery has been associated with increased risks of mortality and postoperative neurocognitive impairment (Watson et al., 2008; Andresen et al., 2014). Moreover, intraoperative EEG suppression is reportedly a predictor of postoperative mortality when it coincides with low mean arterial pressure (MAP) (Willingham et al., 2014). Sessler et al. discovered that the probabilities of longer hospital stay and mortality increased in patients with a “triple low” of low blood pressure, low BIS (higher BSR, lower BIS), and a low minimum alveolar concentration of volatile anesthesia (Sessler et al., 2012). Therefore, detecting the onset of burst-suppression is paramount to preventing the further development of deep anesthesia.

Due to the variations in dose responses among patients to the same anesthetic agent, the maintenance of an appropriate depth-of-anesthesia remains a major challenge to anesthesiologists (Thomsen and Prior, 1996). Monitoring the depth-of-anesthesia during surgery is crucial to the avoidance of intraoperative and postoperative side effects consequent of deep anesthesia. Several studies have shown that some EEG-based monitors for the depth-of-anesthesia feature limitations and lead to a misinterpretation of the level of anesthesia. Even the BIS monitor is reportedly poor at detecting the onset of burst-suppression, and the efficacy of BIS monitoring in guiding the safe reduction of anesthetic doses lacks consistent demonstration (Morledge and Stecker, 2006; Aretha et al., 2008). Hence, prior findings do not provide sufficient support to inform routine monitoring in standard practice. The adequate detection of the latency of deep anesthesia necessary to avoid the adverse reactions caused by the deep anesthesia has thus become a major concern of clinical anesthesiologists.

This study seeks to help to resolve this limitation of clinical practice. We wrote programs in MATLAB to calculate and define the burst-suppression latency (BSL): the time that elapses from the beginning of anesthesia to the first occurrence of burst-suppression. The aims of this study were to further explore the relationship between BSL and the subsequent BSR under isoflurane or adjuvant drugs with isoflurane anesthesia in mice and to find a superior index for detecting the onset of deep anesthesia. By comparing correlation between BSL and BSR of different recording sites, we found the best place to record EEG.

## Materials and Methods

### Animals

Adult (age: 10–12 weeks, weight: 23–25 g), young (age: 3 weeks, weight: 10–12 g) and old (age: 18–24 months, weight: 30–35 g) C57BL/6 male mice were purchased from the Experimental Animal Center of Xuzhou Medical University (Xuzhou, Jiangsu Province, China). Mice were maintained under controlled temperatures and light cycles (24 ± 1 °C, 12/12 h dark/light cycle with lights on at 7:00 am) with ad libitum access to food and water. Under 1.0 and 1.5% isoflurane anesthesia, the mice were divided into three groups including young, adult and old group and the EEG were recorded for 2 h respectively; The adult mice were pretreated with intraperitoneal injection of sub-anesthetic dose of ketamine (25 mg/kg) (Kamiyama et al., 2011), lower doses of dexmedetomidine (15 µg/kg) (Zhang et al., 2015), midazolam (0.5 mg/kg) (Baptista et al., 2009) or propofol (tail vein injection, 5 mg/kg) (Kimura-Kuroiwa et al., 2012) before they were anesthetized by 1.0% isoflurane for 2 h. All procedures were performed in accordance with standard ethical guidelines and were approved by the Animal Care and Use Committee of Xuzhou Medical University and in accordance with the National Institutes of Health Guide for the Care and Use of Laboratory Animals. At the end of the experiments, all mice were euthanized using carbon dioxide asphyxiation under isoflurane anesthesia.

### Stereotaxic Surgery

All mice were anesthetized with 2% isoflurane and mounted on a stereotactic frame (RWD Kopf Instruments, Shenzhen, China) with a Kopf model mouse adaptor. Mice scalps were shaved and sterilized with povidone-iodine, and a 1.0 cm skin rostral-caudal incision was made to expose the skull surface. Tapping screw holes were accomplished with an electric drill. The electrodes were fixed on the skull surface, sealed with dental acrylic (Shanghai New Century Dental Materials Company, China), and attached to a head mount connector for the EEG polysomnographic recording (8403: 0.10″ Screw with Wire Lead Pinnacle Technology, Inc.,KS, United States). The EEG stereotaxic coordinates are as follows: one frontal-electrode, medial-lateral (ML) (−1.0 mm), anterior-posterior (AP) (+1.5 mm); two parietal electrodes, ML (±1.0 mm), AP (−1.0 mm); two occipital electrodes, ML (±1.0 mm), AP (−3.0 mm) (Ren et al., 2018; Wang et al., 2020); and one that was placed on the nasal commissure as a reference electrode. In addition, two Electromyography (EMG) electrodes were placed into the neck muscles of the mice to evaluate muscle movement. All EEG electrodes were plugged into a plastic multichannel electrode pedestal (8235-SM: Surface Mount 6-Pins Connector, Pinnacle Technology, Inc.,KS, United States). EMG electrodes were plugged into another pedestal. Animals were then allowed to recover from surgery for 7 days before the recordings were performed.

### Electroencephalographic Data Acquisition

**FIGURE 1:**
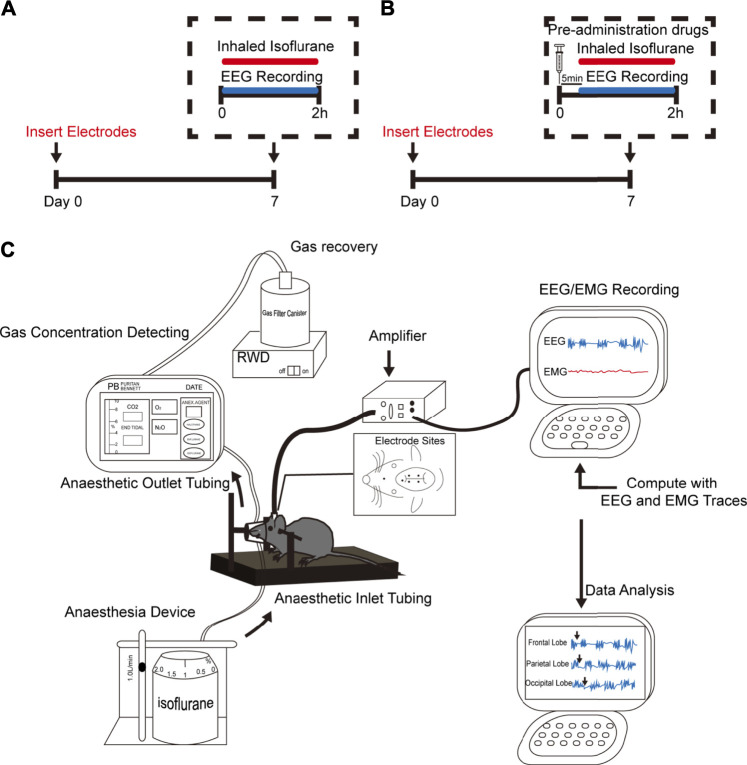
Experimental design. **(A,B)** Schematic timelines illustrating the procedure performed for each experimental animal in this study. The timelines indicate that all animals underwent head-cap surgery 1 week before the experiment. **(A)** Animals only received isoflurane anesthesia. **(B)** Animals received co-administered anesthetics or adjuvant drugs are given 5 min in advance. Throughout the entire experiment, EEG and electromyography (EMG) were performed. **(C)** Schematic shows anesthetic inhalation, EEG/EMG recordings, and data analysis systems. The concentration of administered gas was monitored continuously to ensure the stability of the experimental process.

Figure 1 illustrates the experimental time course. On the day of the experiment, animals were placed into a stereotactic frame with a Kopf model mouse adaptor and connected to the mouse anesthesia mask and the EEG recording system (8202: Mouse Preamplifier Pinnacle Technology, Inc.,KS, United States) (Figure 1C). The mice then received the target concentration of isoflurane anesthesia, the concentration of which was continuously monitored. The EEG/EMG signal was amplified, the EEG was lowpass filtered to 50 Hz, and the EMG bandpass was filtered between 40 and 200 Hz. Data were digitized and sampled at 1,000 Hz using Sirenia software (V1.1.1, Pinnacle Technology, Inc.,KS, United States). During the entire process, the rectal temperature was recorded every 15 min, and a heating pad was used to keep the body temperature constant (±1.0 C).

### Burst-Suppression Latency and Burst-Suppression Ratio

The raw EEG signals were exported into MATLAB (version R2016b, Mathworks, Inc., MA, United States). Signals were digitally filtered and spectrally analyzed by mtspectrumc function to provide information on the frequency, time and amplitude contents in the EEG signals using Chronux2.12 analysis. The parameters were set as follows: parames.Fs (frequency) = 1,000; parames.tapers = [5 9] (5 is the time-bandwidth product and nine is the number of leading tapers to use.); the parameter of movingwin = [2 2] (these represent step and bin, respectively).We calculated amplitude and variance of the first 5,000 samples as the base value. Subsequently observed EEG amplitudes and variance of less than 40% of the base value were considered to indicate a suppression; and of more than 120%, a burst. EEG was shown as a binary time series, and the time that elapses from the beginning of anesthesia to the first occurrence of burst-suppression. In calculation of BSR, we limited the minimum duration of the suppression wave to 0.5 s. The BSR was calculated as the percentage of time spent in suppression of each 1 min binary series. To validate our results, we applied two alternative methods: mean plus three or four standard deviations based on visual inspection and a nonlinear energy operator–based method to calculate the BSR (Hoffman and Edelman, 1995; Sarkela et al., 2002). Further, the detection methods were applied to different channels of EEG signals (monopolar: frontal; bipolar: left–right parietal; left–right occipital).

### Statistical Analysis

Data are presented as the mean ± SD. Statistical analyses were performed with GraphPad Prism 5.0 software (Graph Pad Software, Inc.,CA, United States) and Spss16.0 software package (IBM, Inc.,NY, United States). A correlation analysis between BSL and BSR was performed with Pearson correlation if the data satisfied normality; otherwise, Spearman rank correlation was used. Comparisons between the BSL and BSR of the two groups animals treated with isoflurane alone or in combination with other drugs were performed with an unpaired *t-*test when the variances of the two populations were equal; otherwise, we used a Mann–Whitney U two-sample rank test. Core body temperatures were analyzed with a two-way ANOVA and Sidak multiple comparisons test. Statistical significance was indicated by *p*-values of <0.05.

## Results

### Automatic Recognition and Calculation of Burst-Suppression Latency and Burst-Suppression Ratio

**FIGURE 2:**
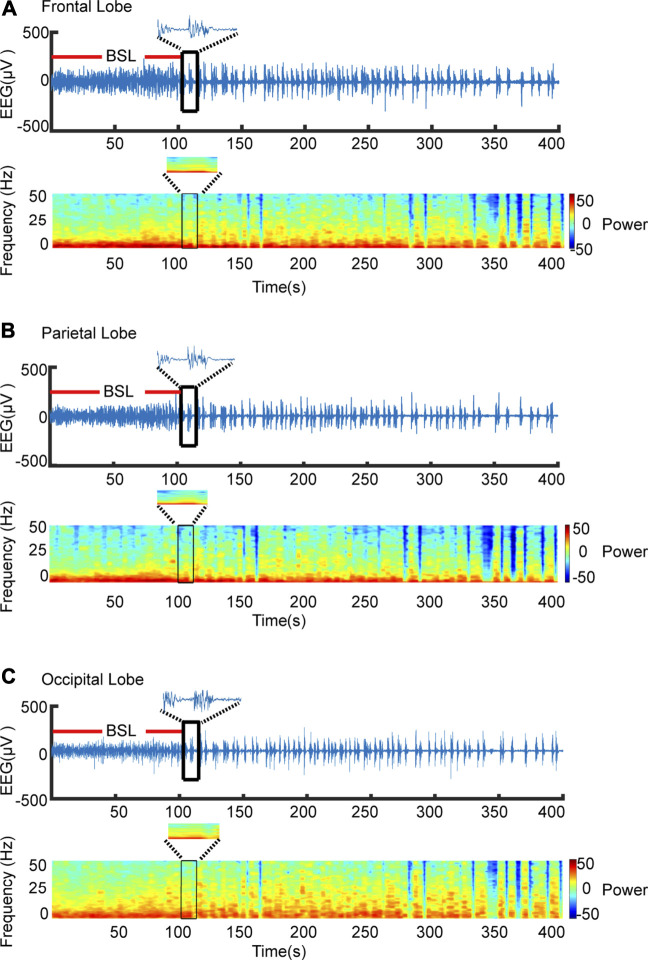
Automatic recognition BSL. **(A)** Representative EEG raw traces **(upper)** and power spectrogram **(lower)** are shown for the occipital lobe. Software recognizes the first burst-suppression as indicated by the black frame. The time from anesthesia induction to the first burst-suppression, where periods of suppression were defined as lasting more 0.5 s, was defined as BSL. **(B**,**C)** Raw and spectrogram maps of the frontal, parietal and occipital, respectively.

Burst-suppression in different brain regions was discriminated automatically by the software. Figures 2A–C shows representative EEG raw traces and power spectrograms for the frontal, parietal, and occipital lobes and indicates the transition into burst-suppression during the deepening of isoflurane anesthesia. The BSL is labeled by a red line in Figures 2A–C, where raw traces indicate the representative alternating pattern of high-amplitude “burst” periods interrupted by sustained low-amplitude “suppression” periods. Despite having performed three different types of analysis to calculate BSR, we found the calculation method of our program shown no differences in BSR from the other two programs. The matching ratio of automatic detection and manual detection was over 87% in BSL.

### Correlation Between Burst-Suppression Latencyand Subsequent Burst-Suppression Ratio Under Isoflurane Anesthesia

**FIGURE 3:**
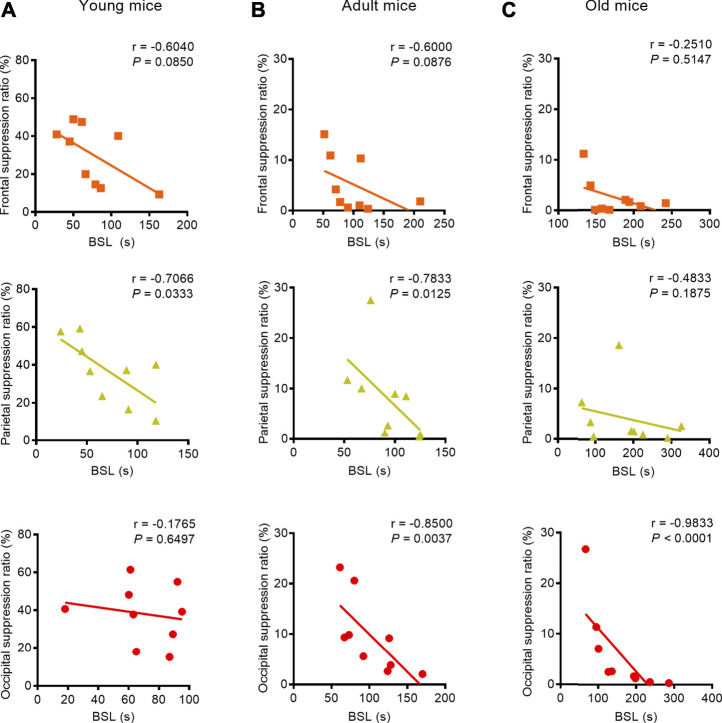
Correlation between BLS and BSR in mice of different ages during the administration of 1.0% isoflurane anesthesia. **(A)** A weak negative correlation between BSL and BSR in the frontal lobe (*n* = 9) and a strong negative correlation in the parietal lobe (*n* = 9) were found in young mice receiving 1.0% isoflurane anesthesia, while the occipital EEG recording indicated no correlation between BSL and BSR (*n* = 9). **(B)** A weak negative correlation between BSL and BSR in the frontal EEG recording (*n* = 9) and strong correlations between BSL and BSR in parietal EEG recording (n = 9) and occipital EEG recordings (*n* = 9) were found in adult mice. **(C)** In old mice, the EEG recording in frontal and parietal lobes showed no correlation between BSL and BSR (*n* = 9, respectively), while a negative correlation was detected in the occipital EEG recording (*n* = 9).

**FIGURE 4:**
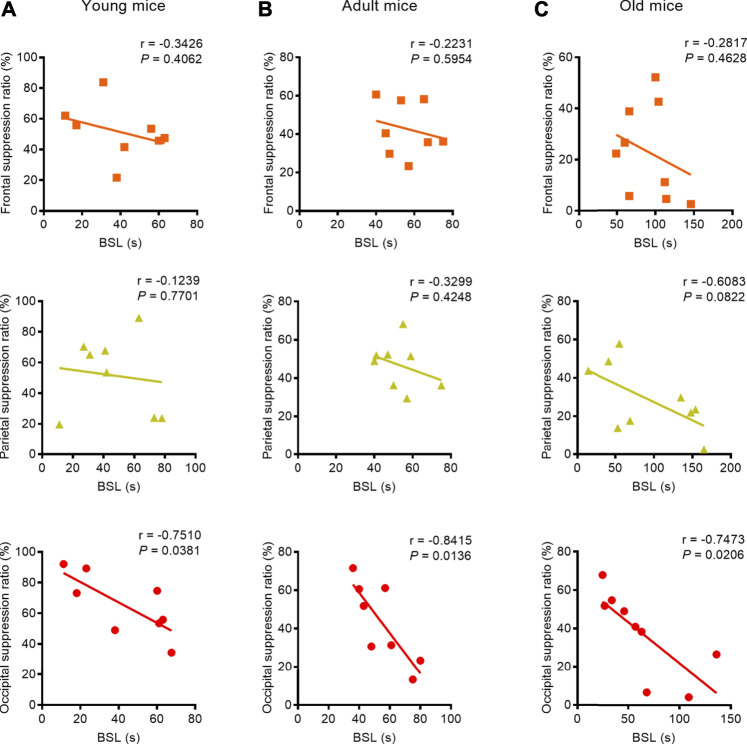
Correlation between BLS and BSR in mice of different ages under 1.5% isoflurane anesthesia. **(A)** Correlation analysis revealed that the BSL was not correlated with BSR in the frontal and parietal EEG recordings in young mice (*n* = 8, respectively), while the occipital lobe EEG recording showed a strong negative correlation (*n* = 8). **(B)** In the adult group, BSL was not correlated with BSR in the frontal or parietal EEG recording (*n* = 8, respectively); only the occipital EEG recording indicated a strong negative correlation between BSL and BSR (*n* = 8). **(C)** In the old group, no correlation was found between BSL and BSR in frontal lobe (*n* = 9), and the parietal showed a weak negative correlation between BSL and BSR (*n* = 9); in the occipital lobe, the correlation analysis revealed strong negative correlation between BSL and BSR (*n* = 9). These results indicate that the occipital lobe may be the best suited for monitoring the depth-of-anesthesia under deep anesthesia.

We found a strong negative correlation between BSL and BSR, especially with respect to the parietal EEG recording in the young mice (parietal: correlation coefficient r = −0.7066, *p* = 0.0333, Pearson correlation), the parietal and occipital EEG recordings in adult mice (parietal: correlation coefficient r = −0.7833, *p* = 0.0125, Spearman correlation; occipital: correlation coefficient r = −0.8500, *p* = 0.0037, Pearson correlation), and the occipital EEG recording in old mice (occipital: correlation coefficient r = −0.9833, *p* < 0.0001, Spearman correlation) (Figures 3A–C). When isoflurane concentration was increased to 1.5%, only occipital EEG recordings showed a negative correlation between BSL and BSR in mice regardless of age (young: correlation coefficient r = −0.7510, *p* = 0.0381; adult: correlation coefficient r = −0.8415, *p* = 0.0136; old: correlation coefficient r = −0.7473, *p* = 0.0206) (Figures 4A–C).

### Change of Burst-Suppression Latency and Burst-Suppression Ratio Under Co-Combination Anesthetic or Adjuvant Drugs With Isoflurane

**FIGURE 5:**
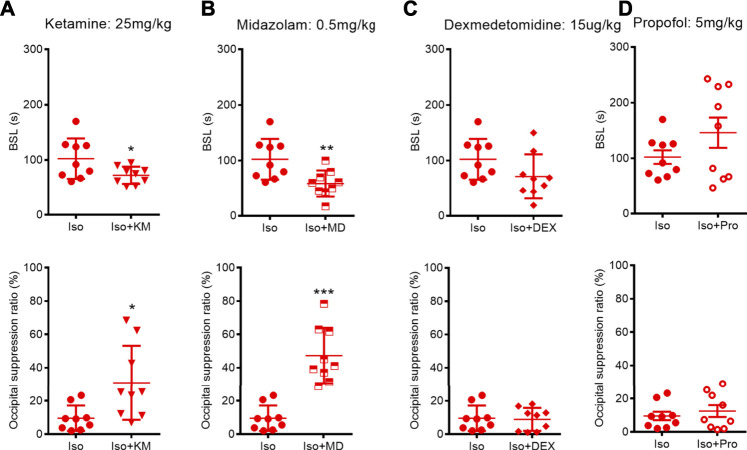
Effects of co-administration anesthetics or adjuvant drugs on BSL and BSR during isoflurane anesthesia. **(A–B)** Animals receiving ketamine or midazolam co-administration showed a significant decreased BSL (*n* = 9) and increased BSR (*n* = 9, respectively) during isoflurane anesthesia. **(C–D)** Dexmedetomidine or propofol co-administration did not change neither BSL nor BSR during isoflurane anesthesia. Data are expressed as mean ± SD, Statistical significance with *p < 0.05, **p < 0.01, ***p < 0.001. Iso, isoflurane; KM, ketamine; MD, midazolam; DEX, dexmedetomidine; Pro, propofol.

In clinical practice, many anesthetics or adjuvant drugs are used in combination; hence, our investigated considered whether the co-administration of these drugs could change the BSL or BSR in adult mice receiving 1.0% isoflurane anesthesia. Combining automatic software analysis with manual analysis, we found that the intraperitoneal injection of a subanesthetic dose of ketamine before that of isoflurane anesthesia caused a significant 29% decrease in the BSL (from 102.3 (36.6) to 72.3 (15.6) s; *p* = 0.0450, unpaired *t*-test) relative to exposure to isoflurane alone. Meanwhile, ketamine administration caused a significant 222% increase in the BSR (from 9.6 (7.6) to 30.9 (22.2) %; *p* = 0.0220, Mann–Whitney *U* test) (Figure 5A). We also found that the co-administration of midazolam not only significantly shortened the BSL (from 102.3 (36.6) to 58.4 (23.8) s; *p* = 0.0082, unpaired *t*-test), but also increased the BSR under isoflurane anesthesia (from 9.6 (7.6) to 47.3 (16.7)%; *p* = 0.0006, Mann–Whitney *U* test). However, the co-administration of dexmedetomidine or propofol (tail vein injection, 5 mg/kg) neither affected the BSL (dexmedetomidine: from 102.3 (36.6) to 71.3 (39.8) s; *p* = 0.1048, unpaired *t*-test; propofol: from 102.3 (36.6) to 146.1 (81.6) s; *p* = 0.1696, a Mann–Whitney *U* test), nor the BSR (dexmedetomidine: from 9.6 (7.6) to 8.9 (6.8)%; *p* = 0.8507; propofol: from 9.6 (7.6) to 12.5 (10.7)%; *p* = 0.5173, unpaired *t-*test; respectively) (Figures 5B,C).

### Correlation Between Burst-Suppression Latency and Subsequent Burst-Suppression Ratio Under the Co-Administration of Anesthetics or Adjuvant Drugs With Isoflurane

**FIGURE 6:**
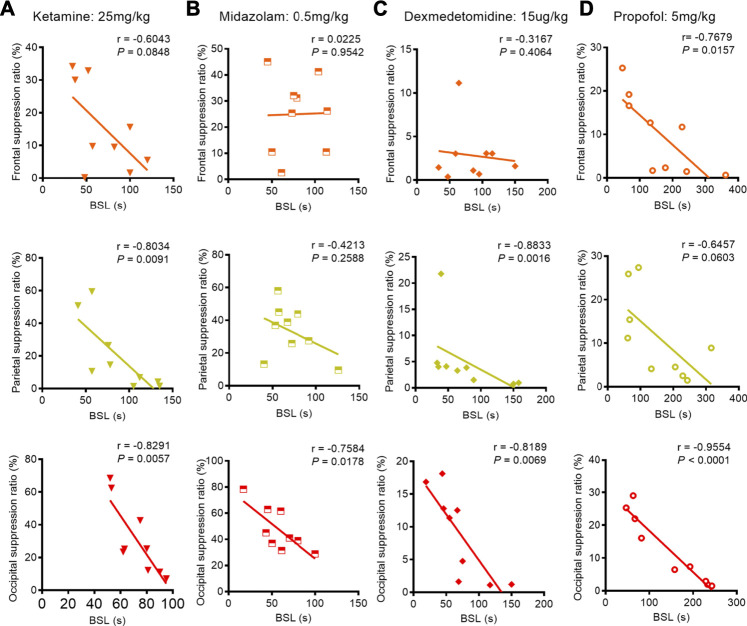
Correlation between BLS and BSR during the co-administration anesthetics or adjuvant drugs with 1.0% isoflurane anesthesia. **(A)** During the co-administration of ketamine with 1.0% isoflurane anesthesia, we found a weak negative correlation between BSL and BSR in the frontal lobe (*n* = 9), while the parietal and occipital recordings revealed strong negative correlations between BSL and BSR (*n* = 9, respectively). **(B)** During the co-administration of midazolam with 1.0% isoflurane anesthesia, no correlation between BSL and BSR was found from frontal or parietal recordings (*n* = 9, respectively); however, the occipital lobe EEG recording indicated strong negative correlation between BSL and BSR (*n* = 9). **(C)** During the co-administration of dexmedetomidine with 1.0% isoflurane anesthesia, no correlation was found between BSL and BSR in the frontal lobe (*n* = 9), while parietal and occipital recordings revealed strong negative correlations between BSL and BSR (*n* = 9, respectively). **(D)** After administering propofol as premedication during 1.0% isoflurane anesthesia, the frontal and occipital EEG recordings showed strong correlations between BSL and BSR (*n* = 9, respectively); however, only a weak negative correlation between BSL and BSR was found from parietal recordings (*n* = 9).

We next studied the correlation between BSL and BSR when adult mice were subjected to 1.0% isoflurane anesthesia with a co-administration of anesthetics or adjuvant drugs. Pearson correlation analysis revealed that the BSL derived from occipital EEG recordings retained a stronger negative correlation with BSR under studied co-administration regimens with isoflurane anesthesia (ketamine: correlation coefficient r = −0.8291, *p* = 0.0057; midazolam: correlation coefficient r = −0.7584, *p* = 0.0178; dexmedetomidine: correlation coefficient r = −0.8189, *p* = 0.0069; propofol: correlation coefficient r = −0.9554, *p* < 0.0001). This correlation was also detected in the parietal EEG recordings when isoflurane was administered with ketamine or dexmedetomidine (ketamine: correlation coefficient r = −0.8034, *p* = 0.0091; dexmedetomidine: correlation coefficient r = −0.8833, *p* = 0.0016) and from the frontal EEG recording when isoflurane was co-administered with propofol (propofol: correlation coefficient r = −0.7679, *p* = 0.0157) (Figures 6A–D).

### Changes in Body Temperature During Experiments

**FIGURE 7:**
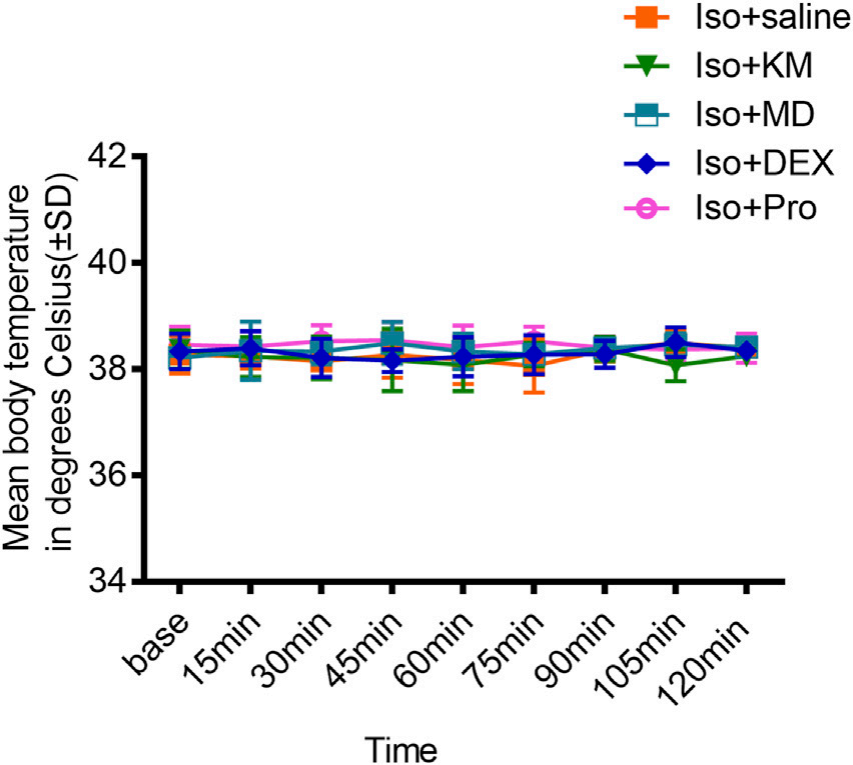
The co-administration anesthetics or adjuvant drugs did not change the body temperature. Comparisons with the saline co-administration group revealed that the core body temperature is not affected by ketamine, midazolam, dexmedetomidine, or propofol co-administration with 1.0% isoflurane anesthesia. (two-way ANOVA: *p* = 0.5883).

As hypothermia might influence BSR, body temperature was continually measured during experiments. A rectal temperature probe was inserted into each mouse, and measurements of body core temperature were taken every 15 min until the animal emerged from anesthesia. No significant changes in core body temperature were found in any of the animals during any of the experiments (*p* = 0.5883; Figure 7).

## Discussion

The data presented in this study demonstrate 1) a correlation between BSL and BSR during isoflurane anesthesia or its co-administration with other anesthetic or adjuvant drugs; 2) that the co-administration of 1.0% isoflurane with ketamine or midazolam, but not with dexmedetomidine or propofol, significantly decreases BSL and increases BSR; 3) a stronger correlation between BSL and BSR in the occipital lobe than in other surveyed regions as anesthesia deepened. These findings may provide a novel index for detecting the onset of and monitoring deep anesthesia.

Some studies have shown that monitoring the depth-of-anesthesia including BIS and BSR may reduce intraoperative awareness and predict long- or short-outcomes in specific high-risk populations (Kent and Domino, 2009; Muhlhofer et al., 2017). However, the currently available depth-of-anesthesia monitors are subject to many limitations, the most significant of which includes the latency between changes in anesthesia and display of the index that diminish the capacity of anesthesiologists to detect and resolve the deep anesthesia in a timely manner (Fahy and Chau, 2018).

BSR is a significant temporal parameter used to quantify the direction of suppression periods and reflect the deep anesthesia (American Society of Anesthesiologists Task Force on Intraoperative, 2006; Koyama et al., 2019). Burst-suppression on an EEG during the administration of anesthesia indicates reduced cerebral activity and deep anesthetic level (Besch et al., 2011; Ching et al., 2013); hence, many depth-of-anesthesia monitors use BSR as an important indicator of deep anesthesia. Burst-suppression has previously been considered a global phenomenon, with synchronous bursts occurring simultaneously across cortical areas (Clark and Rosner, 1973; Dykstra et al., 2012). However, many studies have demonstrated that burst-suppression is highly dependent on local cortical dynamics, as the state evolves both across time and across different cortical areas. Even if the burst-suppression is global, the onset time or duration of burst-suppression varies (Dykstra et al., 2012; Lewis et al., 2013). The present study indeed observed that burst-suppression features spatial heterogeneity. While we found the differences in the BSL and BSR caused correlation biases between BSL and BSR across the cortex and the consistent negative correlation between BSL and BSR calculated from occipital EEG recordings. Therefore, the occipital lobe was identified as the optimal site at which to monitor burst-suppression or deep anesthesia in mice because of the consistent negative correlation between BSL and BSR calculated from occipital EEG recordings.

The accurate detection of the onset of deep anesthesia is further of importance in so far as it maintains the stability of anesthesia and helps the anesthesiologist to avoid placing the patient under an excessive depth-of-anesthesia (Shi et al., 2013). Our study showed that BSL may be used to monitor the deep anesthesia, regardless of whether isoflurane was used alone or in combination with other drugs, specifically, a low BSL was observed to accompany a high BSR and deep anesthesia. Thus, the BSL can help to inform the avoidance of the further development of deep anesthesia which can avoid negative outcomes that bring by deep anesthesia. This is of particular significance to young and old mice, whose developing or aging brains are more vulnerable to the effects of deep anesthesia (Davidson, 2011; Stary et al., 2015; Satomoto and Makita, 2016). Although animal observations cannot be directly applied to humans, these observations could inform the development of a sufficiently robust parameter capable of detecting changes caused by anesthetics on the EEG of different mammals.

The common clinical practice of combining anesthetics could affect the BSL and BSR; indeed, the present study found that the co-administration of ketamine or midazolam, but not dexmedetomidine or propofol, with 1.0% isoflurane anesthesia significantly decreased BSL and increased BSR in adult mice. According to the existing evidence, isoflurane and ketamine have synergistic effects on the observed depth of anesthesia (Hambrecht-Wiedbusch et al., 2017). Similar effects were previously described upon the co-administration of ketamine and propofol (Hui et al., 1995; Albanese et al., 1997). Meanwhile, midazolam belongs to the benzodiazepine class of drugs and functions by increasing the release of the GABA neurotransmitters in the brain (Reves et al., 1985). Previous study indicates that midazolam premedication reduces the requirements for the dose of propofol for multiple anesthetic endpoints, including the attainment of electroencephalographic burst-suppression (Wilder-Smith et al., 2001). Midazolam administration was associated with decreased absolute power for all frequencies of EEG at all electrode sites during isoflurane anesthesia (Keegan et al., 1993). Our results thus indicated that the co-administration of midazolam or ketamine during isoflurane anesthesia may deepen anesthesia.

However, the effect of dexmedetomidine on BSL and BSR differed from that ketamine and midazolam under isoflurane anesthesia. Administering dexmedetomidine, a highly selective a2-adrenergic agonist that selectively activates α2-adrenoreceptors, is an effective way to induce a deep but arousable sedation (Sanders and Maze, 2012). Clinical studies have described spindle (12–16 Hz) oscillations, a key element of human non-rapid eye movement sleep observable on frontal electroencephalogram electrode channels, during dexmedetomidine-induced sedation (Nelson et al., 2003); this finding implies that the dexmedetomidine-induced arousal sedation state shares similarities with normal sleep (Wolter et al., 2006). Since burst-suppression cannot occur in normal sleep, the co-administration of dexmedetomidine does not alter the BSL and BSR during isoflurane anesthesia. Propofol is a short-acting sedative hypnotic that satisfies the requirements for sedative and amnestic effects with limited impact on both the respiratory and cardiovascular system, and reduces accumulation after multiple doses (Deegan, 1992; San-juan et al., 2010). Our study found that the co-administration of sub-anesthesia does of propofol does not deepen isoflurane-induced anesthesia, because low doses of propofol administered alone can increase high frequency power in cortical circuits. The aforementioned results can inform the best method of premedication to avoid the onset of deep anesthesia.

While burst-suppression seems to be a fundamental characteristic of the deeply inactivated brain, it can result from a range of conditions including hypothermia. The fact that core body temperature did not differ between saline- and ketamine-, midazolam-, dexmedetomidine-, propofol-pretreated animals indicates that the reduction of BSL and increase of BSR were not consequent of changes in body temperature while the animals were subjected to isoflurane anesthesia.

In conclusion, our study found that BSL could predict the subsequent BSR in mice that received isoflurane inhalation alone or in combination with ketamine, dexmedetomidine, midazolam, or propofol. Furthermore, the co-administration of ketamine or midazolam was found to decrease the BSL and increase BSR during isoflurane anesthesia. Our findings suggest that, in clinical settings, the anesthesiologist can adjust the administration of the anesthetics according to the BSL and avoid the occurrence of deep anesthesia. We further found that data obtained from the occipital lobe may best inform this practice. However, there are many inconveniences in occipital lobe as anesthesia depth monitoring site. In addition, further work is required to assess the effects of the effects of the interactions of these drug on neural circuits and their relevance to improved perioperative care.

## Data Availability

The original contributions presented in the study are included in the article/Supplementary Material, further inquiries can be directed to the corresponding authors.
